# Inter- and Intrarater Reliability of the Acromioclavicular Joint Injury Classification on Anteroposterior Radiographs

**DOI:** 10.1177/23259671251337470

**Published:** 2025-05-15

**Authors:** Stein Arve Skjaker, Martine Enger, Are Hugo Pripp, Lars Nordsletten, Berte Bøe

**Affiliations:** † Division of Orthopaedic Surgery, Oslo University Hospital, Oslo, Norway; ‡ Institute of Clinical Medicine, University of Oslo, Oslo, Norway; § Oslo Centre of Biostatistics and Epidemiology, Research Support Services, Oslo University Hospital, Oslo, Norway; Investigation performed at Division of Orthopaedic Surgery, Oslo University Hospital, Oslo, Norway

**Keywords:** acromioclavicular joint injury, dislocation, reliability, Rockwood classification

## Abstract

**Background:**

Previous radiographic reliability studies of the acromioclavicular (AC) joint are from smaller and/or selective patient populations.

**Purpose:**

To investigate the inter- and intrarater reliability of the Rockwood classification of AC joint injuries on standard anteroposterior (AP) radiographs in an urban population.

**Study design:**

Cohort study (diagnosis); Level of evidence, 2.

**Methods:**

All suspected shoulder injuries from May 2013 to April 2014 at the Department of Orthopedic Emergency, Oslo University Hospital, were registered by admittance. Patients with anteriosuperior shoulder pain and maximum point tenderness over the AC joint were assessed. In total, 287 consective AC joint injuries were registered of the 2650 shoulder injuries in residents. From the 287 patients with AC joint injury 268 were ultimately included in the present study. On 2 occasions >6 weeks apart and blinded from each other, 2 orthopaedic consultants, 2 orthopaedic residents, and 2 musculoskeletal radiology consultants independently classified the cohort. In the interrater analyses, the raters were also stratified based on clinical experience and specialty. All raters evaluated 2 standardized nonweightbearing AP radiographs for each patient, performed by tilting the beam 15° in the cephalic and caudal direction, respectively. Reliability was assessed based on Cohen kappa (*k*) values.

**Results:**

Interrater reliability for all raters and for the stratified groups in rounds 1 and 2 was substantial for the Rockwood classification. The interrater reliability improved from round 1 to round 2 for all raters (agreement: 92% vs 94%, *k* = 0.66 vs 0.73, respectively), orthopaedic consultants (agreement: 90% vs 93%, *k* = 0.61 vs 0.72, respectively), orthopaedic residents (agreement: 92% vs 94%, *k* = 0.61 vs 0.76, respectively), and musculoskeletal radiology consultants (agreement: 93% vs 94%, *k* = 0.70 vs 0.73, respectively). No significant differences in intrarater reliability between the groups were seen for orthopaedic consultants (*k* = 0.72), orthopaedic residents (*k* = 0.73), and musculoskeletal radiology consultants (*k* = 0.79). One of the radiology consultants had the highest intrarater *k* value (*k* = 0.84), significantly higher than one of the orthopaedic consultants (*k* = 0.67) and one of the orthopaedic residents (*k* = 0.66). The three other raters had substantial intrarater agreement (*k* = 0.75 to 0.80). The raters had a strong majority agreement (≥4/6 raters) of the Rockwood classification in 82% of the patients in round 1, and in 83% in round 2. Rockwood type II had low majority agreement, with no 5- or 6-raters (only ≤4/6) agreement.

**Conclusion:**

Nonweightbearing unilateral AP radiographs of AC joint injuries expressed substantial inter- and intrarater reliability. There was an improvement in interrater reliability for all groups from round 1 to round 2. One of the raters had an almost perfect intrarater reliability, and the rest had substantial reliability. The majority agreement for Rockwood type II injuries was low.

Based on the works by Tossy et al^42^ and Allman^2^ in the 1960s, Rockwood established a detailed classification of acromioclavicular (AC) joint injuries categorized into types I to VI (Table 1).^2,37,42^

**Table 1:** Rockwood Classification of Acromioclavicular Joint Injuries on Radiographs^*a*^

Rockwood Type	CC Distance, %	AC Joint
I	Normal	Normal
II	0-25	Normal or slightly elevated and may be normal or widened AC joint
III	>25-100	Lateral clavicle elevated above superior border of acromion
IV	Increased or normal CCD	Posterocranial displacement of lateral clavicle
V	>100-300	Lateral clavicle elevated grossly above acromion
VI	Reversed CC distance	Inferior AC dislocation

a AC, acromioclavicular; CC, coracoclavicular; CCD, coracoclavicular distance.

Compared with other classification systems for AC joint injuries, the Rockwood classification is the most widely used.^5,30,37,38,42^ Still, there is currently only a consensus-based protocol for the diagnostic evaluation of AC joint injuries.^7,38^ Differences in recommended treatment of AC joint injuries throughout the years may be due to, and perhaps worsened by, the lack of an accurate radiological classification of AC joint injuries.^10,25,36^

There has been an increasing interest in the misdiagnosis of AC joint injuries and changing recommendations for a radiographic and diagnostic approach. In 2014, the Upper Extremity Committee of the International Society of Arthroscopy, Knee Surgery and Orthopaedic Sports Medicine (ISAKOS) suggested a new algorithm of 4 radiologic projections for AC joint injuries.^7^ They suggested a standard anteroposterior (AP) projection, a bilateral Zanca view, an axillary projection, and an additional cross-body adduction view (Basamania/Alexander) to subdivide into stable (IIIA) and unstable (IIIB) type III injuries. Only the AP unloaded radiograph (unilateral Zanca view) or a panoramic view (bilateral Zanca) are the recommended views in a consensus from the European Shoulder Associates (ESA), section of the European Society of Sports Traumatology, Knee Surgery and Arthroscopy (ESSKA).^38^ They dedicated 2 ESA-ESSKA meetings, in 2018 and 2019, to AC joint injuries to initiate high-level research and guidelines, which were published as consensus in 2020.^38^

Studies of different static, dynamic, and loaded radiographs have been published, summarized by Pogorzelski et al^33^ in 2017 and by Aliberti et al^1^ in 2020 on the horizontal instability. Unilateral AP or Zanca radiographs alone have been associated with lower interrater reliability grading AC joint injuries according to Rockwood classification.^30,32^ Kraeutler et al^25^ and Schneider et al^39^ have shown moderate to good agreement in the vertical plane for Rockwood classification, but with some statistical biases. Furthermore, Schneider et al used stress radiographs, which is more challenging in the acute clinical setting. These radiograph reliability studies were all from smaller and/or selective AC joint injury patient populations. Most papers have shown that there are difficulties in detecting horizontal dislocations.^1,8,15,39,41^ Additionally, 3-dimensional computed tomography (CT) scans have not shown any improvement on reliability of classification of AC joint injury, compared with AP and axial radiographs.^10^ Velasquez Garcia et al^43^ found substantial agreement when categorizing AC joint injuries using the modified Rockwood classification by ISAKOS, but the reliability for type IV injuries was worse than by chance. Unilateral nonweightbearing AP (Zanca) radiographs represent a simpler approach in daily clinical practice.

The aim of this study was to investigate the inter- and intrarater reliability of the classification of AC joint injuries on standard nonweightbearing AP radiographs in consecutive patients in a large emergency department. We hypothesized that the Rockwood classification on nonweightbearing unilateral AP radiographs of the AC joint was reproducible between and within raters in a clinical setting.

## Methods

### Ethics

The study was accepted as an internal audit project with anonymous data by the privacy and data protection officer at Oslo University Hospital (OUH). Internal audits are exempt from approval by the regional committee for medical and health research ethics according to Norwegian legislation.

### Study Design and Population

**Figure 1.**
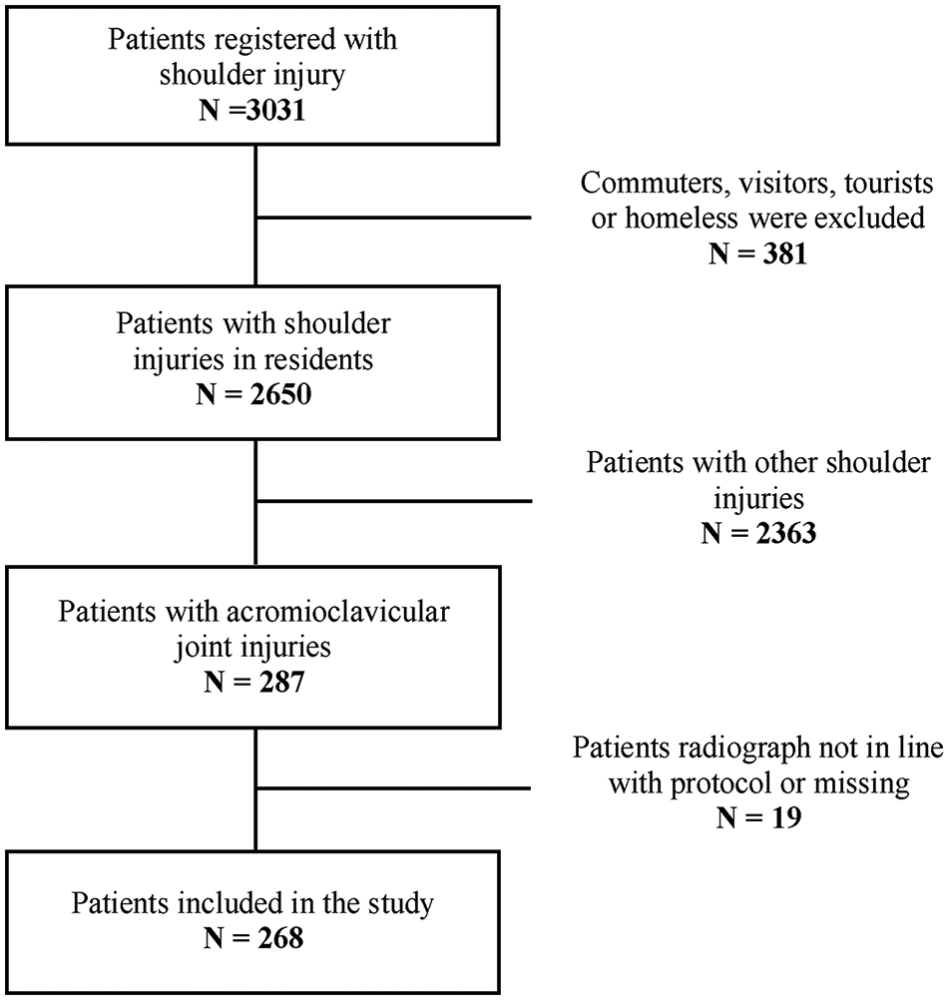
Flowchart of patients.

This was a retrospective cohort study based on prospectively registered data of patients admitted to the Department of Orthopedic Emergency, OUH, from May 2013 through April 2014.^13^ All suspected International Classification of Diseases, Tenth Revision (ICD-10) diagnoses S4 (injury of shoulder and upper arm) were registered on admittance. In addition, all related ICD-10 M-diagnoses were reviewed with the aim of ensuring that patients with acute injury did not mistakenly receive one of these diagnoses. Overall, 3031 patients with shoulder injuries were registered, and 2650 were residents in Oslo (Figure 1). Patients with anterosuperior shoulder pain and with maximal point tenderness over the AC joint were assessed. In total, 287 patients with consecutive AC joint injuries were registered.^40^ We used guidelines for reporting reliability and agreement studies (GRRAS) and a quality appraisal tool for reliability studies (QAREL) to improve our study.^24,28^

### Radiographic Measurements and Classification

**Figure 2.**
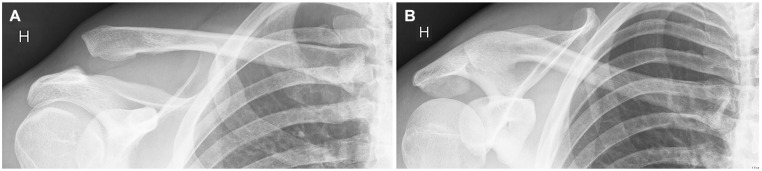
(A) Nonweightbearing anteroposterior (AP) radiograph of the acromioclavicular (AC) joint; a 15° cephalic tilt in the vertical plane. (B) Nonweightbearing AP radiograph of the AC joint in the same patient; a 15° caudal tilt in the vertical plane.

All raters examined 2 standardized nonweightbearing AP radiographs according to the OUH standard protocol, both performed by tilting the x-ray beam 15° each way in the vertical plane (cephalic and caudal tilt), for all patients in a standing position (Figure 2).^9,16,44^

There were both AC and clavicular radiographs, the latter with a slightly more medial focus of the beam. Of all patients, only 11 patients had a panoramic view at the physician's request. They were not used for any of the measurements but were available in the picture archiving and communication system (PACS). Five patients were excluded due to missing radiographs: 2 patients did not have radiographs due to pregnancy, and 3 patients did not want to wait in the Department of Orthopaedic Emergency to have a radiograph taken. An additional 14 patients had a shoulder radiograph not in line with the standard protocol and were consequently excluded from the present analysis. A total of 268 from the 287 patients with clinical suspicion of AC joint injury were ultimately included in the present study.

**Figure 3.**
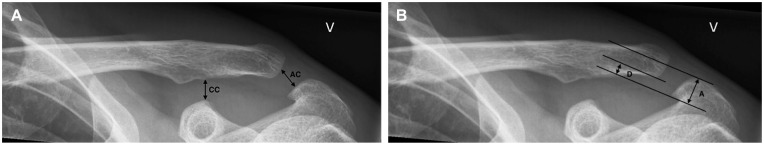
(A) Acromioclavicular (AC) width and coracoclavicular (CC) distance measured for grading according to Rockwood classification. (B) The D/A ratio. A, height of the acromion; D, height distance between the inferior margin line of the acromion and a parallel line through the lowest lateral end of the clavicle. In this case, the ratio is near 0.4, which is equal to a 40% vertical displacement.

The understanding of the measurements and usability of the registration form were tested in a pilot study on 10 patients’ radiographs. After the pilot, more consistent measurements were recommended. For the coracoclavicular (CC) distance, we measured the shortest distance. For the AC width, we measured from the midacromion to midclavicular articular surface. The following was measured in millimeters: the AC width, the CC distance, and the AC height distance (D) between the inferior margin line of the acromion and a parallel line through the lowest lateral end of the clavicle, in caudal and cranial tilt (Figure 3).^17,39^ We also measured the height of the acromion (A) and calculated the D/A ratio described by Gastaud et al.^15^ The D/A ratio was used to guide the raters to an estimate of the percentage of vertical displacement of the distal clavicle relative to the acromion, according to the Rockwood classification.^37^ The CC distance was also measured as an additional indication of dislocation in the vertical plane. A normal CC distance is reported to be 11 to 13 mm, with a wide range of individual variance.^6^ The AC width was mainly used to discriminate between a Rockwood type I and II. The normal AC joint width is reported to be 3.1 ± 0.8 mm in a study of Petersson and Redlund-Johnell^31^ in the frontal plane on AP radiographs. Data also indicate that an AC joint space wider than 7 mm in men and wider than 6 mm in women is a pathological finding.

All measurements were done in the PACS (syngo.via; Siemens Medical) without calibration. We did separate additional agreement analysis with the clavicular and AC joint view. No agreement was defined when <4 raters agreed on the same classification.

#### Ratings

Four orthopaedic surgeons, comprising 2 senior consultants in shoulder surgery (raters 1 (B.B.) and 2 (R.Ø.S.)) and 2 senior (>4 years of training) residents (raters 3 (S.A.S.) and 4), along with 2 musculoskeletal (MSK) radiology consultants (raters 5 and 6) performed the measurements. All 6 raters assessed all 536 radiographs of 268 patients. Repeated measurements were taken after 6 weeks, blinded for the raters’ previous findings. The list of patients was randomized for the second occasion, using https://www.random.org/sequences/. The raters received written instructions on how to perform the required classifications and the radiographic measurements. The clinicians were also blinded for the outcome and completed sessions independently at their own pace. No feedback was provided during or between sessions, and the raters were not allowed to discuss their results.

### Statistical Analysis

All records were used to evaluate inter- and intrarater reliability and agreement. Analyses were performed by stratifying the 6 raters based on their specialty (radiology or orthopaedic surgery) and clinical experience (consultant or senior resident in training for >4 years). Sample size calculations for kappa statistics were performed in Stata with the command *sskdlg*. With an assumption of Cohen kappa (*k*) = 0.8, 6 raters, overall proportion of positive ratings = 0.15, SE = 0.051, and 95% CI, we needed 146 objects.^12^ Rockwood classification data were analyzed using linear weighted *k* for ordinal variables.^11,18,21^ Possible values for *k* statistics ranged from –1 to 1, with 1 indicating perfect agreement, 0 random agreement, and –1 complete disagreement. Cohen *k* was interpreted as follows: almost perfect (>0.80), substantial (0.61-0.80), moderate (0.41-0.60), fair (0.21-0.40), slight (0.00-0.20), and poor (<0.00).^3,26^ We used Stata Version SE 17.0 for analysis (Stata Inc).

## Results

AP radiographs of 268 patients were analyzed. Approximately 25% were high-grade (Rockwood type III or V) injuries (Table 2). In this study, we did not detect any types IV and VI injuries.

**Table 2:** Patients With Acromioclavicular Joint Injury According to Rockwood per Rater on Anteroposterior Radiographs, Round 1 (N = 268)^*a*^

Rockwood Classification Type	Rater 1	Rater 2	Rater 3^*b*^	Rater 4^*b*^	Rater 5^*c*^	Rater 6^*c*^	Mean	Percentage
	Consultant	Consultant	Resident	Resident	Consultant	Consultant		
	Orthopaedic	Orthopaedic	Orthopaedic	Orthopaedic	Radiology	Radiology		
Type I	161	100	178	156	151	155	150.2	56.0
Type II	39	77	37	46	52	42	48.8	18.2
Type III	58	69	43	59	56	57	57.0	21.3
Type V	10	22	10	7	9	14	12.0	4.5

a Data are presented as n unless otherwise indicated. Type IV and VI were not diagnosed with radiographs in this material.

b Orthopaedic residents for >4 years.

c Specialized in musculoskeletal radiology.

### Interrater Reliability

Interrater reliability for all raters in rounds 1 and 2 was substantial for the Rockwood classification (*k* = 0.66 [95% CI, 0.61-0.71] and *k* = 0.73 [95% CI, 0.68-0.78], respectively) (Table 3). For all individual raters and the stratified groups, there was improvement of interrater reliability from round 1 to round 2.

**Table 3:** Interrater Reliability for the Rockwood Classification of Acromioclavicular Joint Injuries for 6 Raters (N = 268)^*a*^

	Round	Percentage of Agreement (95% CI)	Cohen Kappa (95% CI)	Agreement^*b*^
All	1	92 (91-93)	0.66 (0.61-0.71)	Substantial
	2	94 (93-95)	0.73 (0.68-0.78)	Substantial
Orthopaedic senior consultants	1	90 (88-92)	0.61 (0.53-0.68)	Substantial
	2	93 (92-95)	0.72 (0.66-0.79)	Substantial
Orthopaedic senior residents^*c*^	1	92 (90-94)	0.61 (0.52-0.69)	Substantial
	2	94 (93-96)	0.76 (0.68-0.83)	Substantial
MSK radiology consultants^*d*^	1	93 (92-95)	0.70 (0.63-0.78)	Substantial
	2	94 (92-95)	0.73 (0.66-0.79)	Substantial

a MSK, musculoskeletal.

b According to Landis and Koch.^26^

c Orthopaedic residents for >4 years.

d Specialized in MSK radiology.

The raters had a strong majority agreement on the Rockwood classification in 82% of the patients in round 1 and in 83% in round 2 (Table 4). But the majority agreement for type II injuries was poor, as no injury was classified as type II by >4 raters.

**Table 4:** Raters’ Majority Agreement per Rockwood Type for Both Rounds^*a*^

	Agreement: 6-0		Split Agreement: 5-1		Split Agreement: 4-2 or 4-1-1		≥4 Agreement: Total	
	R1	R2	R1	R2	R1	R2	R1	R2
Rockwood type								
I	66	97	59	41	24	14	149	152
II					16	14	16	14
III	10	15	17	12	18	16	45	43
V	3	8	3	3	3	2	9	13
Total	79	120	79	56	61	46	219	222
Proportion of all patients, %	29	45	29	21	23	17	82	83
Strong agreement, ≥5 raters, n (%)	R1: 158 (59)							
	R2: 181 (68)							
Agreement, <4 raters, n (%)							49 (18)	46 (17)
Total							268	268

a Data are presented as n unless otherwise indicated. R, round.

### Intrarater Reliability

The intrarater for one of the radiology consultants was almost perfect (*k* = 0.84), significantly higher than 1 orthopaedic consultant and 1 orthopaedic resident. The orthopaedic surgeons all had substantial agreement (mean *k* = 0.73) for the Rockwood classification (Table 5).

**Table 5:** Intrarater Reliability for the Rockwood Classification of Acromioclavicular Joint Injuries for 6 Raters (N = 268)^*a*^

	Percentage of Agreement (95% CI)	Cohen Kappa (95% CI)	Agreement^*b*^
Orthopaedic senior consultants			
Rater 1	95 (93-96)	0.78 (0.71-0.84)	Substantial
Rater 2	92 (90-93)	0.67 (0.60-0.74)	Substantial
Orthopaedic senior residents^*c*^			
Rater 3	96 (95-97)	0.80 (0.74-0.86)	Substantial
Rater 4	92 (90-94)	0.66 (0.58-0.73)	Substantial
MSK radiology consultants^*d*^			
Rater 5	95 (93-96)	0.75 (0.68-0.82)	Substantial
Rater 6	96 (95-97)	0.84 (0.78-0.89)	Almost perfect

a MSK, musculoskeletal.

b According to Landis and Koch.^26^

c Orthopaedic residents for >4 years.

d Specialized in MSK radiology.

There were no significant differences in inter- and intrarater reliability between clavicular or AC radiographs (Tables 6-9).

**Table 6:** Interrater Reliability of the Acromioclavicular View for the Rockwood Classification of Acromioclavicular Joint Injuries for 6 Raters (n = 109)^*a*^

	Round	Percentage of Agreement (95% CI)	Cohen Kappa (95% CI)	Agreement^*b*^
All	1	92 (90-93)	0.64 (0.55-0.73)	Substantial
	2	94 (92-95)	0.72 (0.64-0.80)	Substantial
Orthopaedic senior consultants	1	89 (87-92)	0.56 (0.42-0.69)	Moderate
	2	95 (93-96)	0.78 (0.69-0.87)	Substantial
Orthopaedic senior residents^*c*^	1	90 (87-94)	0.54 (0.37-0.70)	Moderate
	2	94 (91-96)	0.74 (0.64-0.85)	Substantial
MSK radiology consultants^*d*^	1	93 (91-96)	0.72 (0.59-0.84)	Substantial
	2	93 (91-95)	0.70 (0.59-0.80)	Substantial

a MSK, musculoskeletal.

b According to Landis and Koch.^26^

c Orthopaedic residents for >4 years.

d Specialized in MSK radiology.

**Table 7:** Interrater Reliability of the Clavicular View for the Rockwood Classification of Acromioclavicular Joint Injuries for 6 Raters (n = 159)^*a*^

	Round	Percentage of Agreement (95% CI)	Cohen Kappa (95% CI)	Agreement^*b*^
All	1	93 (92-94)	0.68 (0.62-0.74)	Substantial
	2	94 (92-95)	0.74 (0.67-0.80)	Substantial
Orthopaedic senior consultants	1	91 (89-93)	0.64 (0.55-0.72)	Substantial
	2	92 (90-94)	0.69 (0.60-0.78)	Substantial
Orthopaedic senior residents^*c*^	1	93 (91-95)	0.65 (0.56-0.75)	Substantial
	2	95 (93-97)	0.77 (0.67-0.86)	Substantial
MSK radiology consultants^*d*^	1	93 (91-95)	0.69 (0.60-0.78)	Substantial
	2	94 (92-96)	0.75 (0.67-0.83)	Substantial

a MSK, musculoskeletal.

b According to Landis and Koch.^26^

c Orthopaedic residents for >4 years.

d Specialized in MSK radiology.

**Table 8:** Intrarater Reliability of the Acromioclavicular View for the Rockwood Classification of Acromioclavicular Joint Injuries for 6 Raters (n = 109)^*a*^

	Percentage of Agreement (95% CI)	Cohen Kappa (95% CI)	Agreement^*b*^
Orthopaedic senior consultants			
Rater 1	94 (91-96)	0.72 (0.60-0.84)	Substantial
Rater 2	91 (89-93)	0.62 (0.50-0.74)	Substantial
Orthopaedic senior residents^*c*^			
Rater 3	95 (93-97)	0.77 (0.67-0.88)	Substantial
Rater 4	90 (88-94)	0.62 (0.49-0.76)	Substantial
MSK radiology consultants^*d*^			
Rater 5	95 (93-97)	0.77 (0.66-0.89)	Substantial
Rater 6	96 (94-98)	0.85 (0.77-0.93)	Almost perfect

a MSK, musculoskeletal.

b According to Landis and Koch.^26^

c Orthopaedic residents for >4 years.

d Specialized in MSK radiology.

**Table 9:** Intrarater Reliability of the Clavicular View for the Rockwood Classification of Acromioclavicular Joint Injuries for 6 Raters (n = 159)^*a*^

	Percentage of Agreement (95% CI)	Cohen Kappa (95% CI)	Agreement^*b*^
Orthopaedic senior consultants			
Rater 1	95 (94-97)	0.81 (0.74-0.88)	Almost perfect
Rater 2	92 (90-94)	0.70 (0.62-0.78)	Substantial
Orthopaedic senior residents^*c*^			
Rater 3	96 (95-98)	0.82 (0.75-0.90)	Almost perfect
Rater 4	93 (91-95)	0.68 (0.59-0.77)	Substantial
MSK radiology consultants^*d*^			
Rater 5	94 (92-96)	0.73 (0.64-0.82)	Substantial
Rater 6	96 (94-97)	0.83 (0.76-0.90)	Almost perfect

a MSK, musculoskeletal.

b According to Landis and Koch.^26^

c Orthopaedic residents for >4 years.

d Specialized in MSK radiology.

### No Agreement

For 49 patients in round 1 and 46 in round 2, <4 raters agreed on the Rockwood type (Table 4). It was difficult for the raters to distinguish between Rockwood type I and II in 19 patients in round 1 and 23 in round 2. In 17 patients in round 1 and 6 in round 2, the raters disagreed between type II and III. Twice in round 1 and once in round 2, they were scored equally (3-3) for Rockwood type III or V. Ten times in round 1 and 16 times in round 2, there were other inequalities. Of those with no agreement in round 1, 46% were also lacking majority agreement in round 2.

## Discussion

In this study, inter- and intrarater reliability on AP radiographs was substantial for the Rockwood classification of AC joint injuries in clinical practice. All individual raters as well as the stratified groups improved the interrater reliability from round 1 to round 2. Approximately 25% (68 patients) of this 1-year cohort were Rockwood type III or V injuries. One rater (MSK radiology consultant) had an almost perfect intrarater reliability. The majority agreement for Rockwood type II was very low, but the overall majority agreement for all Rockwood types was >80%. There were no differences in inter- and intrarater reliability for Rockwood classification between clavicular or AC radiographs.

Unilateral AP or Zanca radiographs have been associated with lower interrater reliability when classifying AC joint injuries according to Rockwood classification.^30,32^ Pifer et al^32^ reviewed 50 randomly selected AC joint injuries across 10 years (Rockwood types I-V; excluded IV). AP shoulder radiographs of the patients were investigated by 25 physicians (residents, fellows, and attendings), and the interobserver reliability data were stratified by the physicians’ experience. Our study found a higher reliability of the Rockwood classification on AP radiographs, particularly for the attending MSK radiologist. Pifer et al did not publish data on intrarater reliability.

Ng et al^30^ had 15 surgeons classifying 24 nonweightbearing unilateral AP radiographs (Zanca) according to Rockwood classification, with 9 surgeons participating in the repeated survey. The interrater reliability had a weighted *k* of 0.26 (95% CI, −0.06 to 0.91). The intrarater reliability had a weighted *k* of 0.15 (95% CI, –0.01 to 0.32). Relatively few patients were included, resulting in very wide confidence intervals.

Kraeutler et al^25^ retrospectively identified 28 patients in 42 months who were diagnosed with an AC joint injury type III, IV, or V, with both unilateral AP and axial radiograph. Patients were classified according to Rockwood classification by 1 of 7 fellow surgeon raters based on radiographs, physical examination, and patient history. Interrater reliability of the Rockwood classification was evaluated by the intraclass correlation coefficient (ICC, 0.60; moderate). It is not stated in the article which ICC form was being used.^23,35^

Ringenberg et al^36^ also studied the reliability of the Rockwood classification using unilateral AP and axillary radiographs. They included 50 of 200 identified patients in the study, which may lead to biases. Interrater reliability Fleiss *k* value for the Rockwood classification was 0.27 (fair agreement). Intrarater Cohen *k* value was 0.47 (moderate agreement). The study used Fleiss *k*, which is recommended for studies with randomly selected raters and no connection between raters assessing the various patients.^14,18^

Schneider et al^39^ retrospectively measured 58 patients with bilateral panoramic stress radiographs and an axial radiograph. On 2 occasions, 4 raters independently decided on (1) a visual Rockwood classification of the AC joint injury, (2) a digital measurement of the CC distance and the horizontal dislocation, and (3) a Rockwood classification of the AC joint injury according to the measurements. They found a higher inter- and intrarater reliability of AC joint injuries for the digital measurement in bilateral panoramic stress radiograph (95% CI, 0.85-0.93 and 0.90-0.97, respectively) and axial radiograph (95% CI, 0.62-0.96 and 0.67-0.98, respectively), than for the visual diagnosis (95% CI, 0.72-0.74 and 0.67-0.93, respectively). They used the Pearson correlation coefficient, which has been considered inappropriate and are not recommended for reliability studies.^4^

During a period of 3 years, Cho et al^10^ prospectively included 28 patients. Ten shoulder surgeons analyzed bilateral AP radiographs and an axial radiograph. An additional 3-dimensional CT (taken at the time of initial injury) was presented along with the radiographs 2 weeks later. The interrater and intrarater reliability of the Rockwood classification based on plain radiographs was fair (*k* = 0.21) and moderate (*k* = 0.47), respectively. The additional CT did not improve reliability.

Lau et al^27^ evaluated Rockwood type III and V injuries. They reviewed 55 bilateral Zanca radiographs and found that 6 individual surgeons had substantial agreement in their differentiation between types III and V, with an interrater Fleiss *k* value of 0.62. Three surgeons repeated the process a second time with an intrarater Cohen *k* value of 0.70.

An AC joint injury type II primarily manifests a horizontal instability but may have a slightly increased joint space.^37^ Identifying the horizontal instability of types II and IV can thus be particularly difficult to detect on an AP view. Some studies have tried to assess the reliability of new measurements of horizontal instability with an additional axillary lateral view and dynamic lateral view.^15,39,41^ Tauber et al^41^ introduced glenoacromioclavicular angle (GACA). The interrater reliability of the 3 raters in respect to the GACA was good, with an ICC of 0.82. The intrarater reliability was good with an ICC of 0.78 and 0.81 for 2 raters. Gastaud et al^15^ showed that dislocation in the coronal plane can be analyzed with good reliability using the D/A ratio (tau: 95% CI, 0.69-0.92) and CC ratio (tau: 95% CI, 0.69-0.92), but measurements of horizontal dislocation had large variations. They did not manage to reproduce the promising results of reliability of GACA by Tauber et al.

Velasquez Garcia and Abdo^43^ reviewed the reliability of the suggested Rockwood classification with a IIIA/IIIB (stable/unstable) by ISAKOS, with their recommended 4 sets of bilateral radiographic projections taken at initial injury.^7,43^ The Fleiss *k* value for interrater reliability for the ISAKOS modified Rockwood classification was 0.64 (95% CI, 0.60-0.68), and the Cohen *k* intrarater reliability was 0.62 (95% CI, 0.55-0.64). The subclassification interrater reliability for type IV injuries, despite the extensive radiographic projections, was worse than by chance. The interrater reliability to distinguish between types IIIA/IIIB using the ISAKOS modified Rockwood classification was limited in this study (*k* = 0.22-0.47). The ISAKOS subclassification of IIIA/IIIB is perhaps most useful in a delayed phase and can probably be based on clinical assessment as pain, weakness, decreased range of motion, and a lateral clavicle that dislocates over the acromion in a cross-body test, rather than radiographs.^7^ But special radiographic views (ie, Alexander view) may provide additional information and documentation. Recent studies have come up with 2 new quantitative radiographic parameters that may increase the reliability and validity of the horizontal instability.^22,29,45^

In our study we did not find any significant difference in reliability between clavicular and AC radiographs. Reliability is used to describe a test's random errors. If a test has high reliability, it will show persistent results in several tests.^34^ High reliability is a prerequisite but not a guarantee for validity. It is essential to note that a high reliability in our study for both projections does not mean that they are both equally valid for detecting the true AC joint injury type. In the AC view, the beam is directed more parallel to the joint surface, and it may be assumed that this would provide a more accurate estimate of the AC joint width than the clavicular view. It is our opinion that it is still reasonable to recommend an AC radiograph as the preferred projection, which may also better detect a subtle increase in the joint width indicative of type II injuries.

All raters and the stratified groups improved from round 1 to round 2. This can possibly be explained by the benefit of seeing many AC joint radiographs. Our radiology colleagues were as good as or more reliable than the orthopaedic surgeons in classifying AC joint injuries. Therefore, we still need to discuss AC joint radiographs with radiologists. In our study, we explicitly used the GRRAS guidelines and QAREL checklist for reliability and agreement studies to improve internal validity.^24,28^ To our knowledge, these have never been used for AC joint injury reliability studies before.

The development over recent years has shown a shift in which Rockwood types that are recommended to indicate surgery. We also distinguish between types I and II for recommendations immediately following the injury (ie, avoid heavy lifting). Although most surgeons would not recommend acute surgery for type III, there are still some of those who return for stabilization surgery in the delayed or chronic phase. Some might also recommend the same nonoperative approach for type V as for type III.^19^ We do not yet know if this is the future, as it needs to be further investigated. In our opinion, the Rockwood classification still has a given place in AC joint diagnostics to guide operative and nonoperative treatment.

We believe our findings support that unilateral nonweightbearing AP (Zanca) radiographs have sufficient reliability to be used to categorize vertically displaced AC joints in the acute clinical setting. Our sample size of 1-year consecutive patients from a combined primary care walk-in clinic and secondary care emergency department are more generalizable for the reliability of Rockwood classification of AC joint injuries than most previous findings. Unilateral nonweightbearing AP (Zanca) radiographs also represent an easier implementation in daily clinical practice than 4 radiographs as suggested by ISAKOS and are in line with the suggested radiographs from ESA-ESSKA.^7,38^ Future research is necessary to increase reliability of Rockwood classification in the horizontal plane. We suggest more precise methodological and statistical procedures with limited biases, as well as sufficient methodological information to interpret results.^18^

### Limitations

The study has some limitations. First, this study does not allow us to draw conclusions on the reliability of AP radiographs to detect type IV and VI injuries, as they were not present in the cohort. The incidence of type IV injury varies and is uncertain, but we may have missed some in our material. In Rockwood's 520 cases, only 4 had a type IV injury.^37^ Together with patient history, a clinical examination, and perhaps other radiological examinations, it should be possible to find among 268 consecutive patients. In our study we only assessed AP radiographs, which have shown poor reliability for type IV.^1,33^ Type VI is very rare, often combined with other shoulder girdle injuries, but should be possible to find if present on an AP radiograph.^20^ In combination with AP radiographs and a clinical examination, it should be possible to guide toward a correct AC joint diagnosis.^7^ Second, this study only included radiologic measurements and no clinical findings. The reliability of Rockwood classification of AC joint injuries will likely be better with additional clinical findings. Third, we assessed unilateral images of the injured AC joint and could not determine the percentage of CC distance dislocation compared with the uninjured side. Fourth, there were both AC radiographs and clavicular radiographs, the latter with a slightly more medial focus of the beam. The small difference in the 2 radiographic focuses may decrease the internal validity. On the other hand, the external validity may increase, and our study findings can be generalized to a clinical setting, where both radiographic projections likely will be used. In our data, there were no significant differences between the 2 radiographic projections or any 1-way trends regarding change in agreement.

## Conclusion

In this study, nonweightbearing unilateral AP radiographs of the AC joint had a substantial inter- and intrarater reliability in clinical use. MSK radiology consultants had higher intrarater agreement compared with orthopaedic surgeons. The majority agreement on AP radiographs for Rockwood type II was low. There were no differences in reliability between AC and clavicular radiographs.
